# Occult Follicular Thyroid Carcinoma Presenting as a Frontal Bone Metastasis: A Case Report

**DOI:** 10.1155/2012/678935

**Published:** 2012-02-01

**Authors:** Mahmoudreza Tahamtan, Maral Mokhtari, Sara Pakbaz, Mehdi Tahamtan

**Affiliations:** ^1^Pathology Department, Shiraz University of Medical Science, Shiraz 71937-11351, Iran; ^2^Surgery Department, Shiraz University of Medical Sciences, Shiraz 71937-11351, Iran

## Abstract

This is a rare case of metastatic follicular carcinoma of thyroid in a patient with no history of thyroid problem. A frontal bone mass was examined microscopically and showed the pattern of metastatic follicular carcinoma. Thyroid ultrasonography then revealed a nodule in left lobe. Total thyroidectomy was done for the patient and microscopic examination confirmed the diagnosis.

## 1. Introduction

Follicular thyroid carcinoma (FTC) is the second thyroid malignancy after papillary carcinoma, but compared to papillary carcinoma, it has a greater tendency to distant metastasis to organs such as lung and bone. The lung is the most common metastatic site for thyroid carcinoma followed by bone [1, 2].

The incidence of skull metastasis of FTC is about 2.5 to 5.8% and in most reported cases, metastasis occurred after the diagnosis and treatment of primary tumor; but in few cases, skull metastasis was the first presentation of an occult FTC [3].

Herein, we report a patient with an occult FTC whose initial presentation was a frontal bone mass.

## 2. Case Report

A 42 years old woman presented with a frontal scalp subcutaneous nodule that gradually became larger during 7 months. Her past medical history was unremarkable. Physical examination showed a 6 cm scalp nodule at right frontal area.

**Figure 1:**
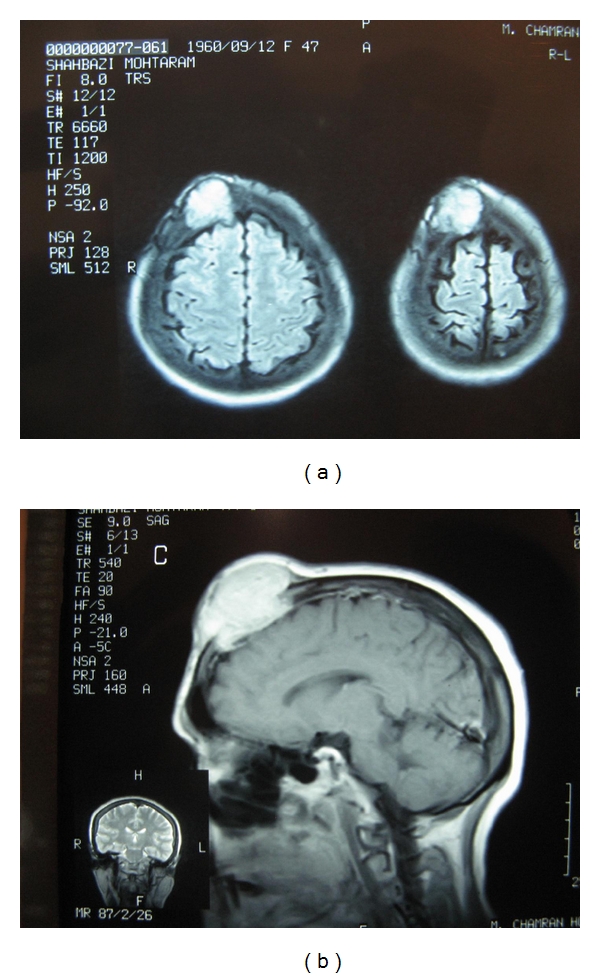
Magnetic resonance image revealed a large destructive mass at right frontal bone with soft tissue invasion.

A brain magnetic resonance image (MRI) and computed tomography (CT) scan were obtained that showed a large lytic lesion arising from right frontal bone with destruction of outer and inner table and diploic space and evidence of soft tissue mass. No calcification was seen in the soft tissue component (Figure 1(a) and 1(b)). The patient underwent surgery. The mass was completely resected with free margins; and the frontal bone defect was reconstructed.

Gross examination of the specimen showed a creamy-brown-colored mass measuring about 6 cm in diameter rested on a fragment of bone. Bone invasion was evident in step sectioning.

**Figure 2:**
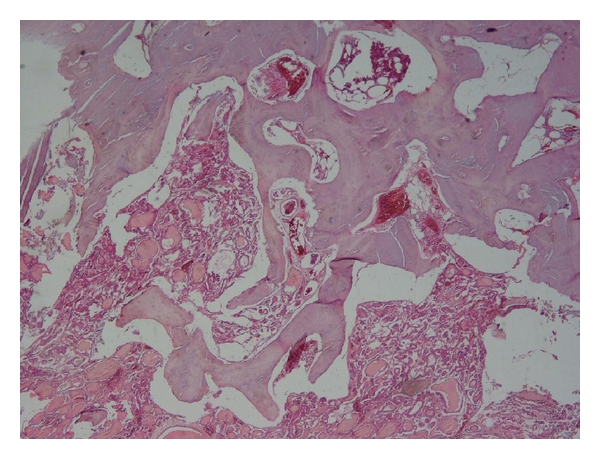
Follicular thyroid carcinoma with skull metastasis. The microfollicles are invaded bone trabecula. Hematoxylin and eosin stain, ×40.

Histologic study showed multiple well-formed microfollicles lined by single layer of cuboidal cells with centrally located nuclei. Invasion of bone trabeculae by the follicles was confirmed on microscopic study (Figure 2).

 Follicular cells were immunoreactive for cytokeratin and thyroglobulin.

The patient was asked for history of any thyroid problem. She did not mention any history of thyroid enlargement, pain, or other symptoms of thyroid disease. Thyroid examination was normal.

Thyroid function test showed normal hormone levels. Then, thyroid ultrasonography was done which revealed a well-defined hyperechoic mass in deep part of left lobe, suspicious for malignancy. So, total thyroidectomy was performed.

**Figure 3:**
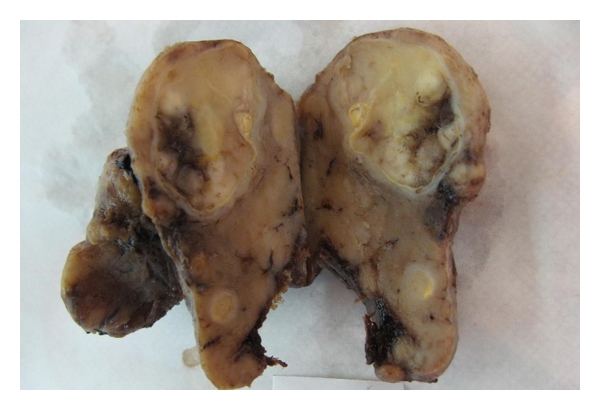
Thyroid gland with enlargement of left lobe. There are two well-defined creamy-gray nodules at the left lobe.

Gross examination of the thyroid showed enlargement of left lobe. Multiple cut sections revealed two well-defined creamy-gray nodule in left lobe of thyroid. The larger nodule is measuring about 3 cm and the other is measuring about 0.5 cm in greatest diameter (Figure 3). 

**Figure 4:**
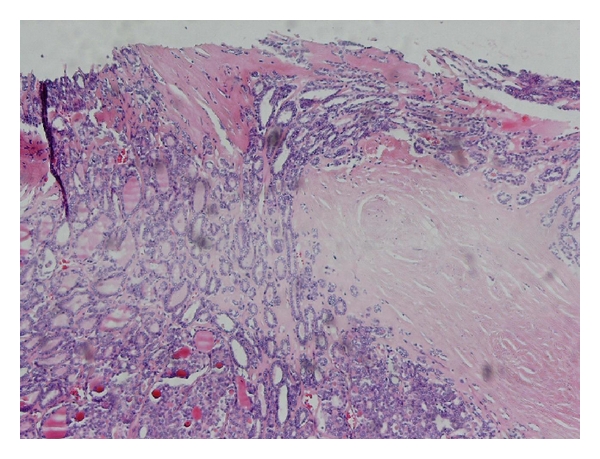
Microscopic study of thyroid gland nodule which shows a follicular carcinoma with area of capsular invasion. Hematoxylin and eosin stain, ×100.

**Figure 5:**
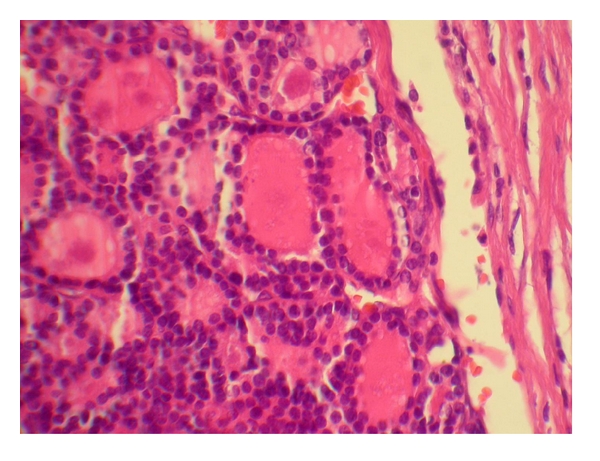
Thyroid follicular carcinoma forming follicles with uniform small nuclei. Hematoxylin and eosin stain, ×400.

Microscopic examination of the nodule showed a follicular neoplasm with various, sized microfollicles and area of capsular invasion; nuclear features of papillary carcinoma (intranuclear inclusions, grooves, and ground-glass appearance) were absent. Representative sections (10 sections) show no vascular invasion (Figures 4 and 5). So, the patient diagnosis was follicular thyroid carcinoma with skull metastasis.

## 3. Discussion

Thyroid carcinomas are the most common endocrine cancers, the prevalence of which is about 5% of thyroid nodules [4]. The most common type is papillary carcinoma and the second is follicular carcinoma [1, 2, 5, 6].

Follicular carcinoma, when compared to papillary carcinoma, occurs in older patients, has hematogenous spread rather than lymphatic, is more aggressive [6, 7], and FTC has a higher propensity to have distant metastasis at presentation [8]. Lung and bone are the most common sites of metastasis of FTC. 1% to 3% of all well-differentiated thyroid carcinomas (papillary and follicular) metastasize to bone [6, 9].

Bone metastasis from FTC is often to ribs, vertebra, and sternum. Skull is a rare site for metastasis of FTC. In most reported cases, skull metastasis of FTC were located in the skull base and occipital area [1], but in our case, it was seen at the frontal bone.

The treatment of choice of follicular carcinoma is total thyroidectomy with radioiodine administration and TSH-suppressive therapy [5].

Five-year survival for stage IV FTC is less than 50% compared to 95% for patients with tumor confined to the thyroid gland. So thyroid examination and early detection and evaluation of thyroid nodules may help to diagnose thyroid cancer before distant metastasis occur [10].
